# Circulating *Mycobacterium tuberculosis* DosR latency antigen-specific, polyfunctional, regulatory IL10^+^ Th17 CD4 T-cells differentiate latent from active tuberculosis

**DOI:** 10.1038/s41598-017-10773-5

**Published:** 2017-09-20

**Authors:** Srabanti Rakshit, Vasista Adiga, Soumya Nayak, Pravat Nalini Sahoo, Prabhat Kumar Sharma, Krista E. van Meijgaarden, Anto Jesuraj UK J., Chirag Dhar, George D. Souza, Greg Finak, Stephen C. De Rosa, Tom H. M. Ottenhoff, Annapurna Vyakarnam

**Affiliations:** 1 Laboratory of Immunology of HIV-TB co-infection, Centre for Infectious Disease Research, Indian Institute of Science, Bangalore, India; 2 Department of Infectious Diseases, Leiden University Medical Center, Leiden, The Netherlands; 3 Department of Infectious Diseases, St John’s Research Institute, Bangalore, India; 4 Department of Pulmonary Medicine & Department of Infectious Diseases, St John’s Research Institute, Bangalore, India; 5 Vaccine and Infectious Disease Division, Fred Hutchinson Cancer Research Center, Seattle, WA United States of America; 6 Department of Laboratory Medicine, University of Washington, Seattle, WA United States of America; 7 Dept. Infectious Diseases, School of Immunology & Microbial Sciences, Faculty of Life Sciences & Medicine, King’s College London, Guy’s Campus, London, SE19RT United Kingdom

## Abstract

The functional heterogeneity of T cell responses to diverse antigens expressed at different stages of *Mycobacterium tuberculosis* (Mtb) infection, in particular early secreted versus dormancy related latency antigens expressed later, that distinguish subjects with latent (LTBI), pulmonary (PTB) or extrapulmonary (EPTB) tuberculosis remains unclear. Here we show blood central memory CD4 T-cell responses specific to Mtb dormancy related (DosR) latency, but not classical immunodominant secretory antigens, to clearly differentiate LTBI from EPTB and PTB. The polyfunctionality score integrating up to 31 DosR-specific CD4 T-cell functional profiles was significantly higher in LTBI than EPTB or PTB subjects. Further analysis of 256 DosR-specific T-cell functional profiles identified regulatory IL10 ^+^ Th17 cells (IL10^+^IL17A^+^IL17F^+^IL22^+^) to be significantly enriched in LTBI; in contrast to pro-inflammatory Th17 cells (IFNγ^+^IL17A^+^/IL10^−^) in the blood and lung of EPTB and PTB subjects respectively. A blood polyfunctional, Mtb DosR latency antigen specific, regulatory, central memory response is therefore a novel functional component of T-cell immunity in latent TB and potential correlate of protection.

## Introduction

Tuberculosis (TB) remains one of the world’s deadliest communicable diseases^1^. Emergence of multi (MDR) or extensively (XDR) drug-resistant forms of *Mycobacterium tuberculosis* (Mtb), coupled with the lack of effective vaccines, absence of clear correlates of protection and accurate diagnostics to classify the diverse clinical stages of TB severely compromises control of the global TB epidemic^2^. The vast majority of infected subjects (~90%) contain infection in a sub-clinical dormant stage known as latent TB infection (LTBI); only ~10% of immunocompetent infected individuals develop active, contagious TB during their lifetime^3^. Active TB can clinically manifest as either pulmonary TB (PTB) or extrapulmonary TB (EPTB). EPTB constitutes about 15–20% of all TB cases but accounts for 50–60% of cases in HIV co-infected immunocompromised individuals^4^. The primary site of PTB is the lung parenchyma, whereas EPTB, which occurs in isolation or along with a pulmonary focus, can manifest in lymph nodes (tuberculous lymphadenitis which accounts for 35% of EPTB), pleura, abdomen, genitourinary tract, skin, joints, bones, meninges and other organs. The diagnosis of extrapulmonary TB remains challenging, involving invasive fine needle aspiration (FNA) and biopsy collection. Further, sensitivity of acid-fast bacilli (AFB) smears are often low due to the paucibacillary nature of the disease^5^. Importantly, the major drawback of the Interferon Gamma Release Assay (IGRA) is its inability to differentiate between healthy subjects latently infected with TB, PTB and EPTB. Although predicted to be different^6^, a definitive analysis of the distinctive features of T cell immunity in PTB, EPTB and latent TB is lacking. We addressed this issue using advanced flow cytometry to dissect the Mtb-antigen specific T cell response in clinically well-defined EPTB, PTB and LTBI subjects from India.

An effective antigen-specific CD4 T cell response is critical for TB control and maintaining a disease free state^7–9^, with loss of CD4 T cells in HIV infection remaining the single most important driver of active TB incidence globally^10,11^. Murine models of TB have highlighted IFNγ and TNFα to be particularly important. IFNγ gene knock-out mice are more susceptible to infection^12^ and neutralising TNFα promotes active TB^13^. MIP1β-deficient MTB-specific CD4 T cells from HIV-infected subjects are preferentially depleted which leads to reactivation of tuberculosis^10^. Recent studies have also emphasized the role of Th17 cells in TB, which have originally been identified as important in mucosal immunity and front line defence in preserving gut epithelial integrity^14^. Vaccination of Mtb-infected mice elicits Th17 cells that secrete chemokines (CXCL9, CXCL10 and CXCL11) that recruit IFNγ^+^CD4^+^ T cells to the infected lung associated with bacterial clearance/control^15–17^. Moreover, adoptive transfer of Mtb-specific Th17 cells conferred protection upon Mtb challenge^18^. However, a definitive description of Mtb-specific cells in humans is lacking. In the blood, Mtb-specific polyfunctional CD4 T cells simultaneously expressing IFNγ and IL2^19^ or IFNγ, IL2, and/or TNFα can correlate with TB latency^20–23^, while other studies found this particular functional profile^24,25^ as well as single Mtb-specific TNFα^+^ cells^26^ to be associated with TB and disease severity. Similarly, despite a role for Th17 effectors in protective immunity^27,28^, other studies have attributed elevated circulating Th17 numbers^29^ and higher proportions of CD4^+^IFNγ^+^IL17^+^ T-cells in blood and pleural fluid from low responding subjects with active TB, to poor clinical outcome^30^. Recently Arlehamn *et al*., demonstrated that 10 kDa culture filtrate protein (CFP10)p52–66/HLA-DRB5*01:01 tetramer-specific CCR6^+^CXCR3^+^CCR4^−^ memory Th1/Th17 cells producing IFNγ, IL2 and TNFα but not IL17, were significantly increased in LTBI donors^31^.

We hypothesized the lack of a definitive functional profile of specific CD4 T cells in TB to be a consequence of two experimental factors: (i) considering subjects with clinically diverse disease states, specifically EPTB and PTB subjects, as representative of a single disease group^26^, and (ii) restricted knowledge of the functional composition of the T cell response to the vast majority of T cell antigens expressed by Mtb during various stages of its life cycle, with most studies focussing on a few secretory antigens expressed early in infection^20–26^. To test this hypothesis, we carefully segregated LTBI, PTB and EPTB subjects on established clinical criteria and probed the diversity of their T cell response using 16-colour advanced multicolour flow cytometry to a panel of eight Mtb antigens expressed early versus late after infection^32^. Specifically, we probed T cells specific for DosR (Rv3133c) regulon encoded (latency) antigens which are expressed late after infection. The DosR regulon comprises of at least 48 genes which can be induced *in vitro* by hypoxia, low-dose nitric oxide and carbon monoxide; conditions encountered by Mtb *in vivo*, when persisting in immunocompetent hosts^33,34^. Many studies have found Mtb DosR regulon encoded antigens to be preferentially recognized by T cells from LTBI as opposed to TB patients^35–37^. Here, T cell responses to four of these antigens were compared with responses to secreted antigens, including antigens belonging to cell wall and cell processes functional category, which include well studied members of either the ESAT-6 family including ESAT-6, CFP10 and TB10.4 or the mycolyl-transferases of the Ag85 complex. These 4 secretory antigens are immunodominant, crucial for Mtb virulence, and the focus of most studies^38,39^. Our comprehensive analysis demonstrates for the first time that CD4 T cell responses to latency, but not secretory TB antigens, clearly differentiate between the three clinical groups of LTBI, PTB and EPTB subjects. Furthermore, our data highlights functional significance of DosR-specific polyfunctional regulatory IL10^+^ Th17 cells to be a novel component of T-cell immunity in latent TB.

## Results

### Subjects with pulmonary and extrapulmonary TB differ markedly in CD4 T cell responses to Mtb latency, but not classical immunodominant secretory antigens

**Figure 1:**
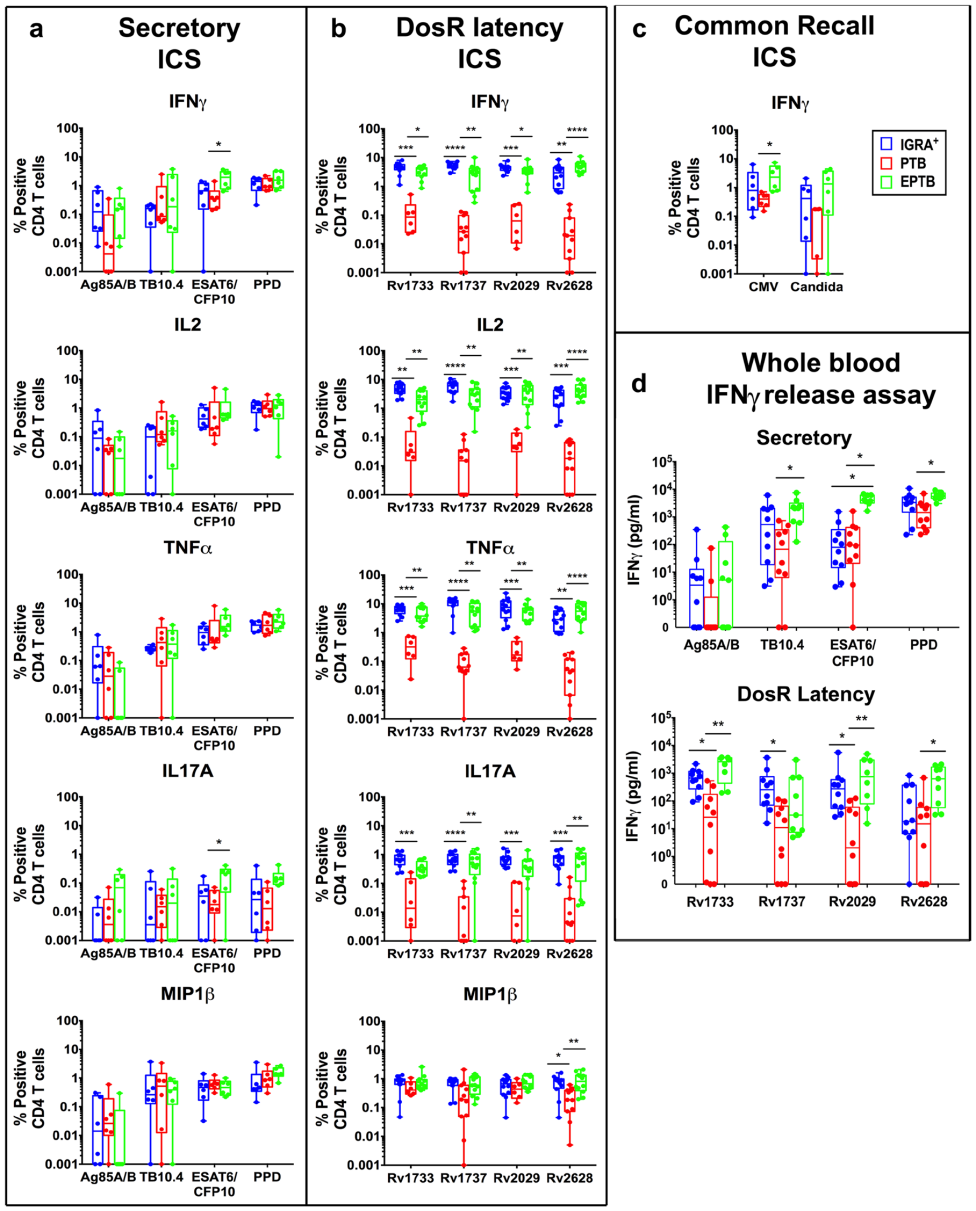
CD4 T cell frequencies to Mtb-specific and common recall antigens. PBMCs from subjects with LTBI (blue), PTB (red) and EPTB (green) were cultured overnight in a standard ICS assay in the presence or absence of (**a**) secretory (Ag85A/B, TB10.4, ESAT6/CFP10 & PPD), N = 6 donors for each group, (**b**) DosR latency (Rv1733c, Rv1737c, Rv2029 & Rv2628), N = 12 donors for LTBI and EPTB group and N = 6 or 12 donors for PTB group and (**c**) common recall (CMVpp65 & CandidaMP65) antigens, N = 6 donors for each group. CD3^+^CD4^+^ T cells were analysed for intracellular expression of IFNγ, TNFα, IL2, IL17A and MIP1β. Box-and-whisker plots show the range in frequencies of total cytokine-positive CD4^+^ T cells with the horizontal bar within the box showing the median. (**d**) IFNγ ELISA was performed on supernatants collected after seven day stimulation of diluted heparinized blood from IGRA^+^ (N = 10), PTB (N = 10) and EPTB (N = 8 or 9) subjects with secretory and DosR antigens. Box-and-whisker plots represent the minimum and maximum IFNγ levels (pg/ml) on a log-10 scale . The line within the box shows the median. Data was analysed using Non-parametric One-Way ANOVA Kruskal-Wallis test and Dunn’s multiple comparisons test. Significant differences are indicated: *p < 0.05; **p < 0.01; ***p < 0.001; ****p < 0.0001.

The Mtb-specific CD4 T cell response in IGRA^+^, PTB and EPTB subjects was analysed for expression of 5 key cytokines: IFNγ, IL2, TNFα, MIP1β and IL17A by multiparameter flow cytometry. Figure 1 shows total frequencies of cytokine expressing CD4 T cells to stimulation by four secretory antigens in panel a (Ag85A/B, TB10.4, ESAT6/CFP10 and PPD); 4 DosR (latency) antigens in panel b (Rv1733c, Rv1737c, Rv2029 and Rv2628) and 2 common recall antigens in panel c (CMV, Candida). Representative FACS plot depicting the stepwise gating strategy to identify functional subsets is shown in Supplementary Fig. 1. All three clinical groups had comparable CD4 T cell responses to secretory antigens, with the exception of ESAT6/CFP10 stimulation, which induced significantly lower IFNγ and IL17A in PTB compared to EPTB subjects (Fig. 1a). The total frequency of IFNγ^+^ CD4 T cells to ESAT6/ CFP10 and PPD stimulation in latent TB was similar in our study to that previously reported^21^. On the other hand, responses to all four latency antigens were significantly compromised in PTB vs. EPTB and LTBI subjects across four cytokines tested (IFNγ, IL2, TNFα, IL17A), consistent with some other studies^35–37^, with EPTB subjects having similar frequencies of specific cells as LTBI (Fig. 1b). An important distinguishing feature was the ability of latency but not secretory antigens to efficiently induce CD4 T cell IL17A in both LTBI and EPTB subjects (Fig. 1a,b). There was a tendency for responses to the common recall antigen, CMV, to also be compromised in PTB subjects but this was not consistent across all cytokines tested (Fig. 1c, IFNγ only shown). Noticeably, we did not detect significant CD8 T cell responses to any of the secretory antigens across all clinical groups; however two latency antigens, Rv2029 and Rv1737c, induced high frequencies of IFNγ, IL2 and TNFα secreting CD8 T cells in latent and EPTB subjects (Supplementary Fig. 2), in line with a previous study^36^. A high throughput 7-day whole blood IFNγ release assay further confirmed that PTB responses to Mtb DosR antigen stimulation is compromised. Thus, latency antigens Rv1733c, Rv2029 and Rv2628 induced significantly higher IFNγ in EPTB vs. PTB subjects. The secretory antigens, TB10.4 and ESAT6/CFP10 also induced higher IFNγ in EPTB compared to PTB (Fig. 1d). Differences in the capacity of cell-associated versus secreted antigens to activate T cells may be linked to (a) antigen processing efficiency, (b) differences in antigen availability /concentration and /or (c) differences in cell trafficking – secreted antigens may activate cells continuously and promote better cell trafficking and egress.

Taken together, we demonstrate clear differences between PTB and EPTB subjects. PTB subjects have lower frequencies of latency antigen specific CD4 T cells in blood and this difference can also be captured by a high throughput whole blood IFNγ release assay, whilst EPTB subjects have similar total frequencies of CD4 T cells as LTBI.

### CD4 T cell memory phenotype differs markedly between latent TB versus active disease

**Figure 2:**
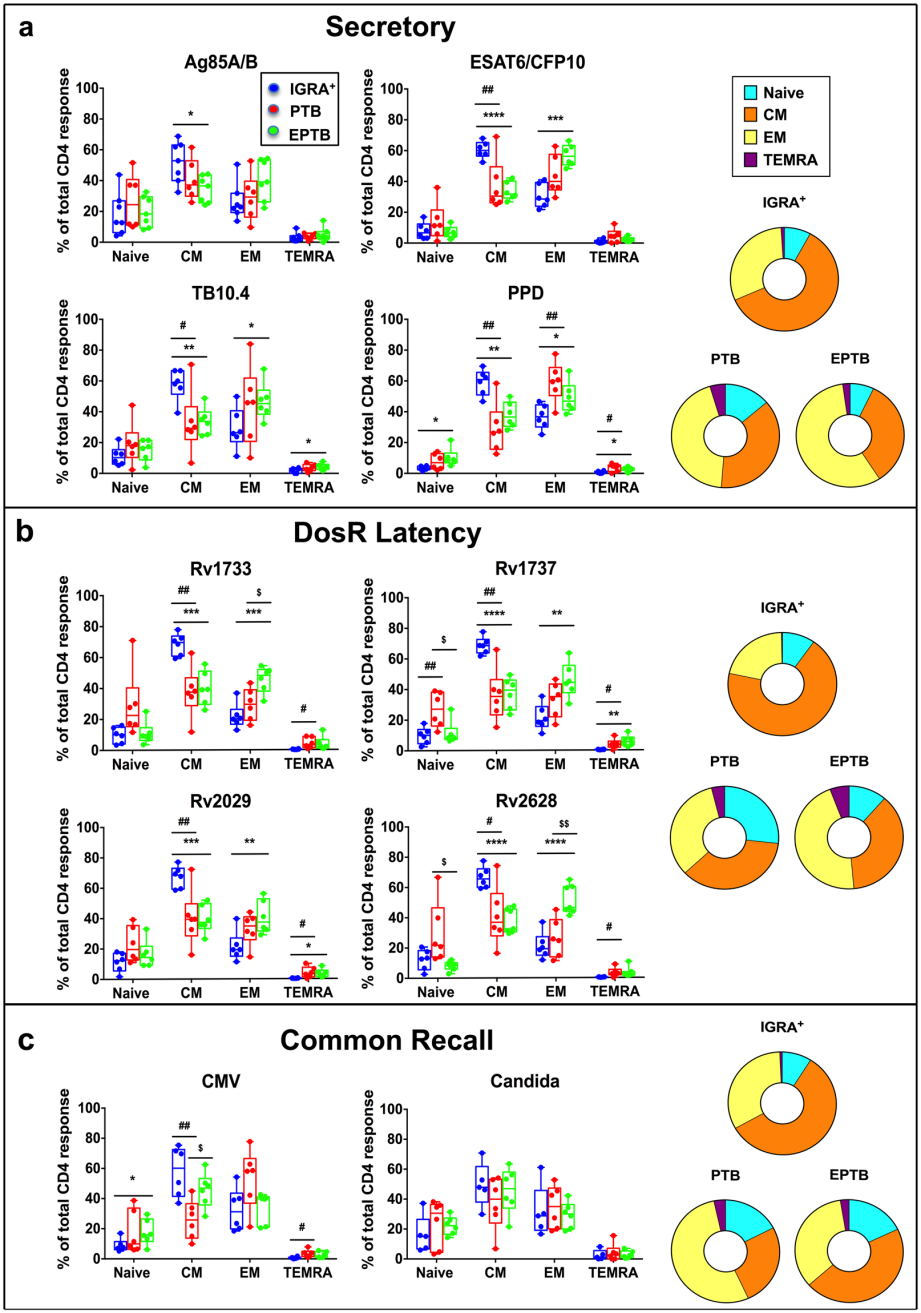
Subjects with latent TB versus disease differ in composition of antigen-specific CD4 T cell memory response. PBMCs from subjects with LTBI, PTB and EPTB (N = 6) were cultured overnight in a standard ICS assay in the presence or absence of (**a**) secretory, (**b**) DosR and (**c**) common recall antigens. The percentage of antigen-specific CD4 T cell subsets expressing IFNγ^+^ and/or IL2^+^ was analysed for CD45RA and CD27 expression to distinguish: naïve (CD45RA^+^CD27^+^), central memory (T_CM_, CD45RA^−^CD27^+^), effector memory (T_EM_, CD45RA^−^CD27^−^) and terminally-differentiated T effector memory cells expressing CD45RA (TEMRA, CD45RA^+^CD27^−^). Box-and-whisker plots show the range in percentages of IFNγ^+^ and/or IL2^+^ memory subsets with the horizontal line within the box showing the median. Each colour slice in the donut graphs correspond to the average percentage of a specific memory subset. One-Way ANOVA was used to determine statistical significance between groups. *p < 0.05; **p < 0.01; ***p < 0.001; ****p < 0.001 (IGRA^+^ vs EPTB), ^#^p < 0.05; ^##^p < 0.01 (IGRA^+^ vs PTB), ^$^p < 0.05; ^$$^p < 0.01 (EPTB vs PTB).

Although the frequencies of specific total cytokine positive CD4 T cells did not differ between controlled infection (LTBI) and disease (EPTB) (Fig. 1), we hypothesized that this response will functionally differ in terms of CD4 T cell memory composition and polyfunctionality, given the marked differences in bacterial burden between the two groups. We predicted that subjects with higher bacterial burden, due to persistent antigen stimulation would have higher effector than central memory responses. Two markers, CD45RA and CD27, that distinguish naïve (CD45RA^+^CD27^+^), central memory (T_CM_, CD45RA^−^CD27^+^), effector memory (T_EM_, CD45RA^−^CD27^−^) and terminally-differentiated T effector memory cells expressing CD45RA (TEMRA, CD45RA^+^CD27^−^) were used. Figure 2 displays the memory phenotype of IFNγ and/or IL2 Mtb-specific CD4^+^ T cells. A similar pattern of memory responses was noted to all ten stimuli tested, with EPTB and PTB subjects consistently having significantly higher effector memory cells than LTBI and conversely, LTBI subjects consistently having significantly higher central memory cells compared to PTB and EPTB subjects (Fig. 2a–c) (p < 0.01 to most stimuli with the exception of Ag85A/B). Whilst these were the dominant memory subsets, differences in TEMRA and naïve specific cells were also observed in a more antigen-dependent manner. TEMRA were detected in EPTB upon stimulation with latency antigens Rv1737c and Rv2029, whereas very low frequencies of this subset were seen in IGRA^+^ subjects to all antigens, however, this was not always significantly lower than TB groups. Thus, the composition of the memory response identifies both groups of subjects with disease (PTB and EPTB) to be distinct from those without disease (latent infection).

### Latent TB subjects induce higher polyfunctional, Mtb latency antigen specific CD4 T-cell responses than subjects with disease

Although previous studies have reported on polyfunctional Mtb secretory antigen specific T cell responses^38,39^, ours is the first study to comprehensively compare such responses in LTBI versus EPTB and PTB subjects to both secretory and latency Mtb antigens.

**Figure 3:**
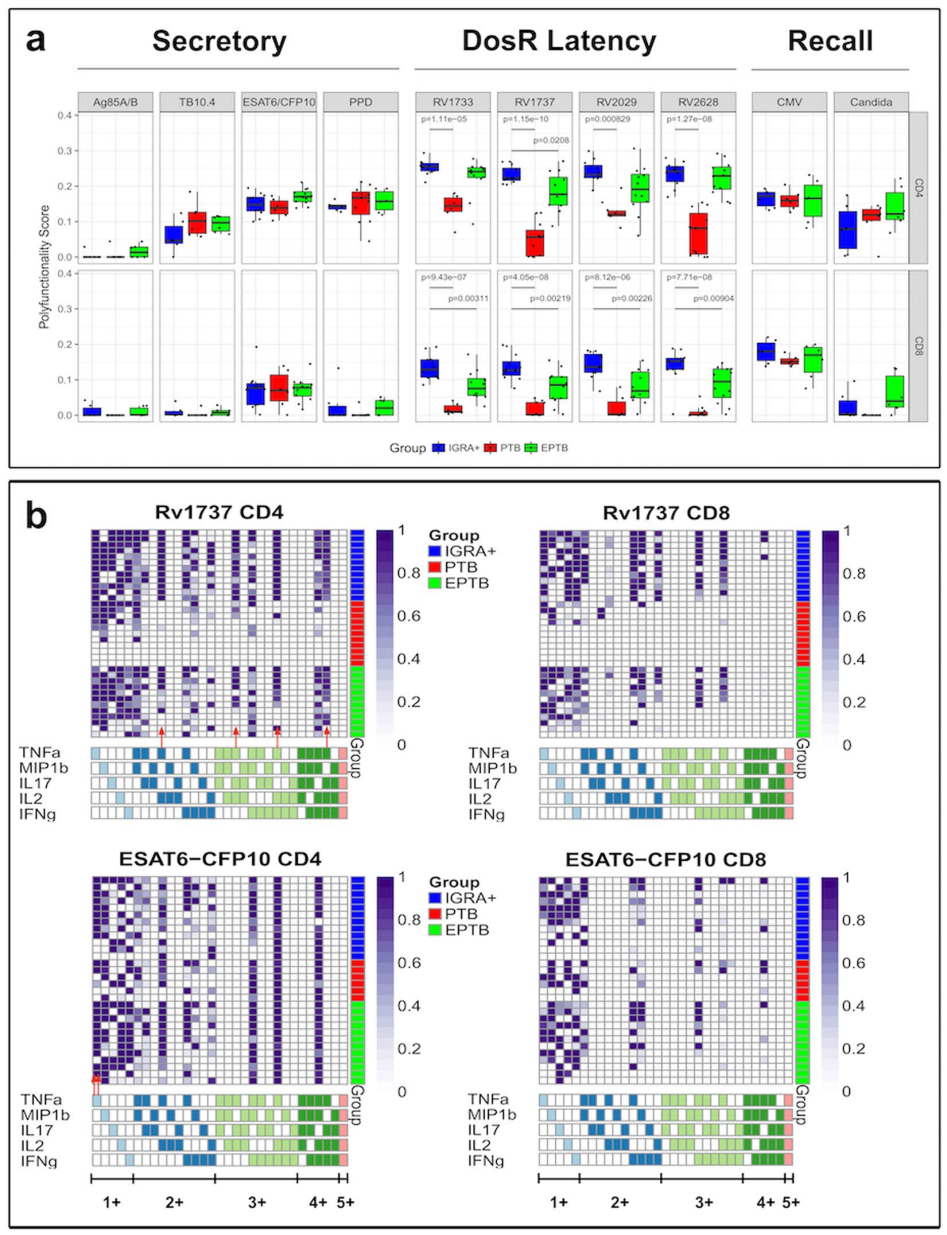
COMPASS analysis reveals enhanced polyfunctional responses to latency antigens in CD4 and CD8 T cells from IGRA^+^ subjects compared to EPTB and PTB patients. (**a**) Box plots of polyfunctionality scores in IGRA^+^ (blue), PTB (red), and EPTB (green) in response to secretory (Ag85A/B, TB10.4, ESAT6/CFP10 & PPD), DosR (Rv1733c, Rv1737c, Rv2029 & Rv2628) and common recall (CMVpp65 & CandidaMP65) antigens. For each antigen, pairwise differences between IGRA^+^ and other groups were based on a group wise linear model fit to the PFS (null: ${\hat{\beta }}_{group}-{\hat{\beta }}_{IGRA+}=0$ two-sided test) and p-values adjusted for multiple testing (see methods). (**b**) Stacked COMPASS heat maps displaying CD4^+^ and CD8^+^ T cell responses to latency antigen Rv1737c and ESAT6/CFP10 in three clinical groups. In the heat map, columns correspond to the different disjoint cell subsets in which responses were detected and are color-coded by the cytokines they express (white = “off”, shaded = “on”, grouped by color = “degree of functionality”), and are displayed in order of increasing functionality from left to right (sky blue to peach). For example, the first column represents CD4 T cells that produce TNFα but none of the other functions. Rows represent study subjects (N = 12 per group), which are ordered by their status: IGRA^+^ (top), PTB (middle) and EPTB (bottom), and by PFS within each group. Each cell of the heatmap shows the probability estimated by COMPASS that the observed response is antigen-specific in the corresponding subject (row) and cell subset (column), where the probability is color-coded from white (zero) to purple (one). A probability of 0 indicates certainty that the observed response is background, while a probability of 1 indicates certainty that the observed response is antigen-specific.

Data in Fig. 1 and Supplementary Fig. 2 was analysed using COMPASS^40^ to enumerate antigen-specific polyfunctional responses of total CD4 and CD8 T cells, respectively, analysing all 31 possible combinations of IFNγ, IL2, TNFα, IL17A and MIP1β. Polyfunctional responses of each subject in each group to each of the ten antigens tested is summarized through the COMPASS polyfunctionality score (PFS) (Fig. 3a). Differences in PFS between groups were estimated through a linear model fit to the PFS for each antigen (see Methods). After multiple testing adjustments, significant differences were found only for the four Mtb DosR latency antigens, for both the CD4 and CD8 T cell subsets. PTB subjects had lower average polyfunctionality than IGRA^+^ subjects for all DosR antigens in their CD4 and CD8 T cell responses. Rv1737c was the only antigen to reveal clear differences in CD4 T cell polyfunctionality response between IGRA^+^ and EPTB subjects, with the former having a higher PFS score, and Rv2029 showing a similar trend (Fig. 3a). On the other hand, the CD8^+^ T cell response PFS score to all four latency antigens was significantly higher in IGRA^+^ compared to EPTB subjects (Fig. 3a).

Heatmaps of cell-subset specific response probabilities in all subjects in three clinical groups studied (Fig. 3b: each row is one donor) for Rv1737c specific CD4 T cell responses (which differed between IGRA^+^ vs. EPTB – see Fig. 3a) versus ESAT6/CFP10 (which did not) is shown. The data demonstrate the increased Rv1737c polyfunctional response in CD4^+^ T-cells of IGRA^+^ compared to EPTB subjects which include combinations of 3 and 4 cytokines, encompassing IL17-containing CD4 T cell subsets. These IL17-containing polyfunctional responses are largely absent in the CD4^+^ T-cells seen in the PTB group, and entirely absent in the CD8 T cell compartment across all groups. Therefore, the number of functional subsets induced by Rv1737c was lower in the CD8 compartment. Most IGRA^+^ subjects responded to Rv1737c and had higher probabilities of 2^+^ (TNFα and IL2), 3^+^ (IL17A, IL2 and TNFα; IFNγ, IL2 and TNFα) and 4^+^ (TNFα, IL17A, IL2 and IFNγ) subsets unlike EPTB where only 50% subjects were positive for these subsets (single arrow, Fig. 3b). Our data, unlike a previous report^26^, did not identify ESAT6/CFP10 specific single TNFα^+^ positive CD4 T cells to be higher in subjects with pulmonary or disseminated extrapulmonary TB from latent TB (two arrows, Fig. 3b) attributed possibly to the segregation of EPTB and PTB subjects into two distinct groups in our study.

### The Th17 response to latency antigens further distinguishes LTBI from EPTB subjects

Th17 cells can potentially play opposing roles in immunity to infection, with cells producing IL17 and IL10 considered to play a protective regulatory homeostatic role and Th17 cells expressing IFNγ in the absence of IL10 being associated with pathology^30,41^. Whether TB infection alters the Th17 subset balance in humans is currently unknown. As latency but not secretory antigens clearly induced IL17 in EPTB and LTBI subjects (Fig. 1, panel b), we probed this response further using an extended ICS panel optimised to detect Th17 subsets comprising of eight cytokines, in an additional set of clinical samples.

**Figure 4:**
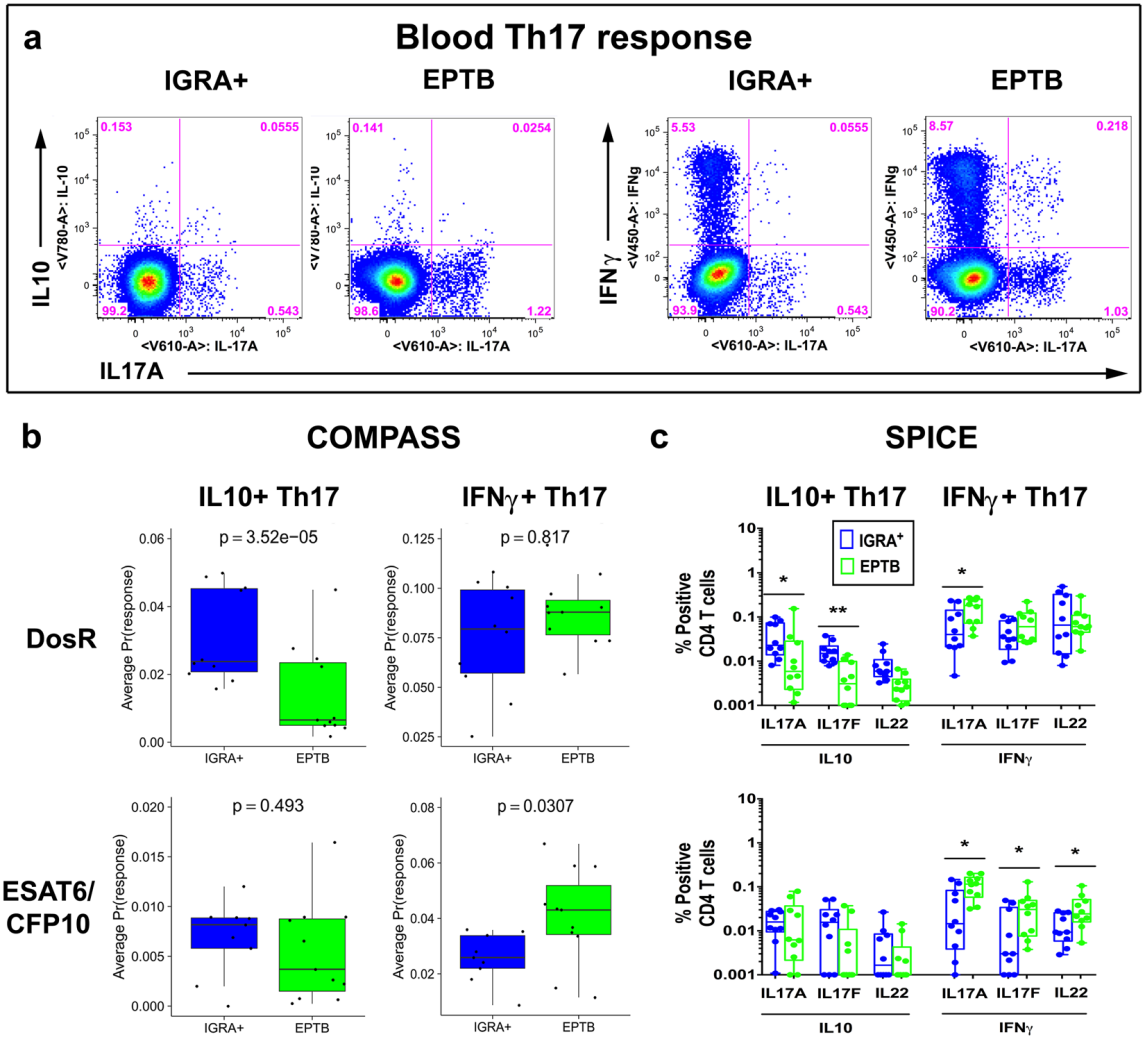
Th17 responses to latency antigens differ between IGRA^+^ individuals and EPTB subjects. PBMCs from subjects with LTBI and EPTB (N = 10) were cultured overnight in a standard ICS assay in the presence or absence of DosR (Rv1733c, Rv1737c, Rv2029 & Rv2628) antigens and ESAT6/CFP10 fusion protein. CD3^+^CD4^+^ T cells were analysed for intracellular expression of IFNγ, IL17A, IL17F, IL22, IL10, TNFα, IL2 and MIP1β (**a**) Representative IL10 versus IL17A and IFNγ versus IL17A flow cytometry graphs in an IGRA^+^ and an EPTB subject to Rv1737c stimulation. Boolean gates were created from the 8 individual cytokine (listed above) gates in FlowJo to divide responding cells into 256 distinct subsets corresponding to all possible combinations of these functions and this data analysed using COMPASS (**b**) or SPICE software (**c**). (**b**) Boxplots of data is shown for the EPTB and IGRA^+^ groups of probability scores of antigen-specific response for each subject, averaged across selected functional profiles (either IFNγ^+^, or IL10^+^ cells together with all combinations of IL22, IL17F and IL17A) and across DosR antigens, or for ESAT6/CFP10. Probabilities are derived from a COMPASS model fit to ICS data using the Th17 panel. A p-value is shown tested for a difference between groups (two-sided t-test). (**c**) Box-and-whisker plots show the range in frequencies with horizontal bar representing the median frequencies of specific double positive Th17 subsets averaged across all 4 DosR antigens or ESAT6/CFP10 stimulation analysed by SPICE. Statistical differences between groups was calculated by Mann-Whitney U test: *p < 0.05; **p < 0.01.

Representative flow cytometry graph (Fig. 4a) shows the staining profile and gating for cells single and double positive for IL10/IL17A vs IFNγ/IL17A following DosR antigen stimulation. Both COMPASS (Fig. 4b) and SPICE (Fig. 4c) software was used to probe this data set. COMPASS was used to analyse antigen-specific polyfunctional responses of total CD4 T cells expressing all 256 combinations of IFNγ, IL2, TNFα, MIP1β, and IL10 along with Th17 cytokines IL17A, IL17F and IL22. Probability of antigen-specific response for each subject averaged across selected functional profiles for either IFNγ^+^ or IL10^+^ together with all combinations of IL22, IL17F and IL17A to all four DosR antigens versus ESAT6/CFP10 is summarized in Fig. 4b. Differences between groups were estimated through a linear model fit for each antigen (see Methods). The probability of CD4 T cell responses to ESAT6/CFP10 and DosR antigens differed between IGRA^+^ and EPTB subjects, with the former having a significantly higher probability for DosR specific cell subsets expressing IL10/IL22/IL17A/IL17F (regulatory Th17), whereas the latter have a higher probability for secretory antigen specific cell subsets expressing IFNγ/IL22/IL17A/IL17F (pathogenic Th17). A similar pattern of difference between the clinical groups to ESAT6/CFP10 versus DosR antigen stimulation was noted using SPICE^42^ (Fig. 4c). SPICE analysis further revealed the regulatory IL10^+^ Th17 response to DosR antigens to comprise higher frequencies of IL10^+^IL17A^+^ and/or IL10^+^IL17F^+^ but not IL10^+^IL22^+^ in IGRA^+^ compared to EPTB subjects. These analysis did not reveal a difference in single IL10 frequencies between the two groups (data not shown), which may be linked to differences in cell source. IL10 single positive cells can include both T effectors and Tregs, whereas IL10/IL17 co-expression is likely exclusively from regulatory IL10^+^ Th17 effector cells. COMPASS data for each DosR antigen tested separately show regulatory IL10^+^ Th17 cells to be significantly higher in IGRA^+^ vs EPTB for Rv1733c and Rv2029, with a similar trend for Rv1737﻿c﻿ (Supplementary Fig. 3a). This is supported by SPICE analysis (Supplementary Fig. 3b) showing Rv1733c and Rv2029 specific IL10^+^IL17A^+^ and IL10^+^IL17F^+^ to be the most significantly different between IGRA^+^ vs EPTB subjects; differences between these groups to Rv1737c and Rv2628 stimulation was weaker.

Thus, our results for the first time reveal the presence of a significantly higher DosR latency antigen specific regulatory IL10^+^ Th17 cells in subjects with latent TB compared to subjects with extrapulmonary TB disease. Conversely, the response to secreted immunodominant antigen ESAT6/CFP10 was skewed to a proinflammatory IFNγ^+^ Th17 response in subjects with extrapulmonary disease.

### Presence of Mtb latency antigen specific pro-inflammatory Th17 cells in the lung of PTB confirms this subset to be a marker of subjects with disease

**Figure 5:**
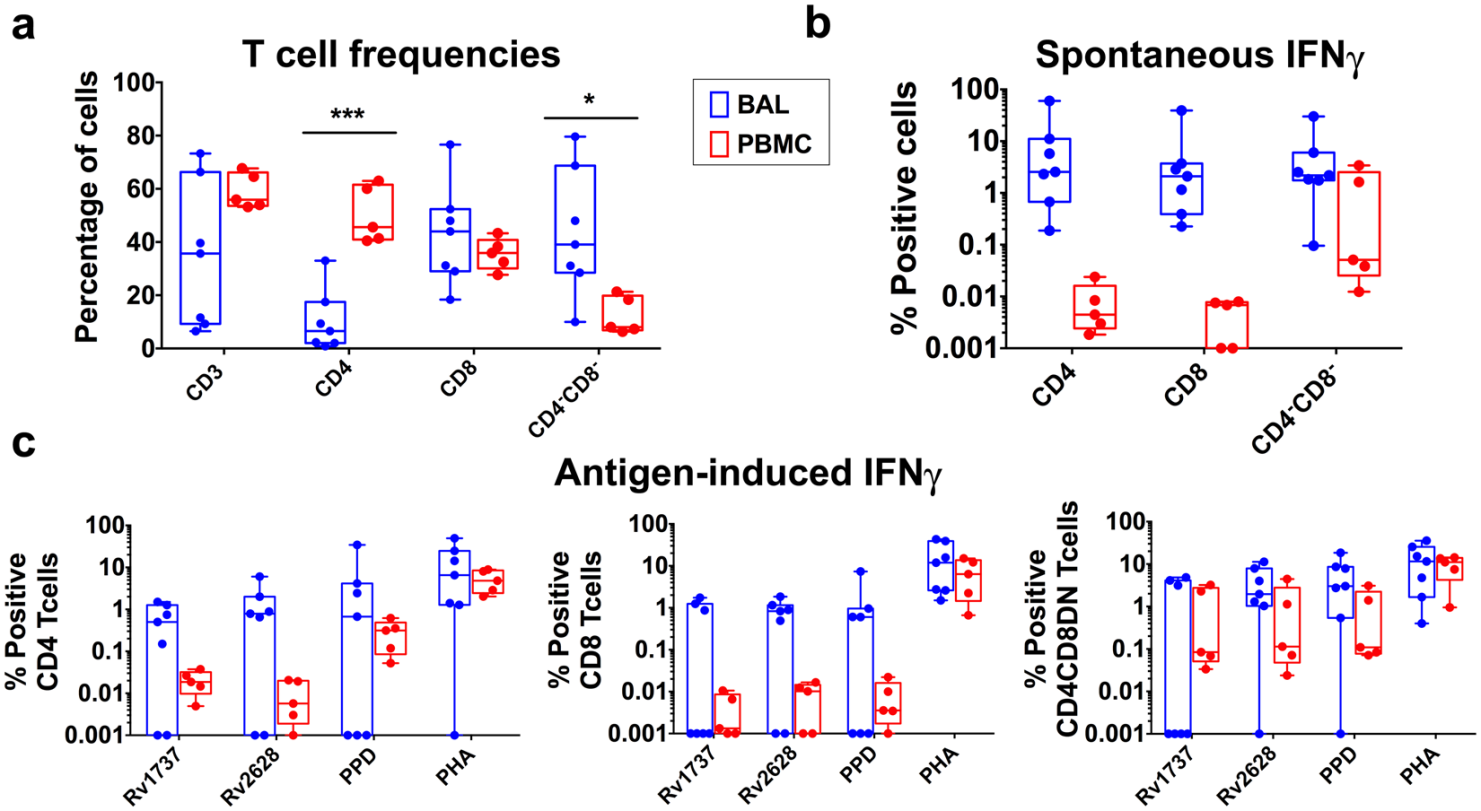
Comparative analysis of immune cells in BAL versus PBMC of PTB subjects. (**a**) Percentage of adaptive subsets within the live lymphocyte gate in BAL (blue) and matched PBMC (red) of PTB patients (N = 7). (**b**) Box-and-whisker plots show the range in frequencies with horizontal bar representing the median of spontaneous IFNγ expressing cells in BAL (blue) and matched PBMC (red). (**c**) Box-and-whisker plots show the range in frequencies with horizontal bar representing the median of antigen-induced IFNγ expressing cells (after subtracting spontaneous release) in BAL (blue) and matched PBMC (red). Difference between BAL and PBMC were analysed using nonparametric Wilcoxon matched-pairs signed rank test and corrected for multiple comparisons with Sidak-Bonferroni test in GraphPad Prism. *p < 0.05; ***p < 0.01.

**Figure 6:**
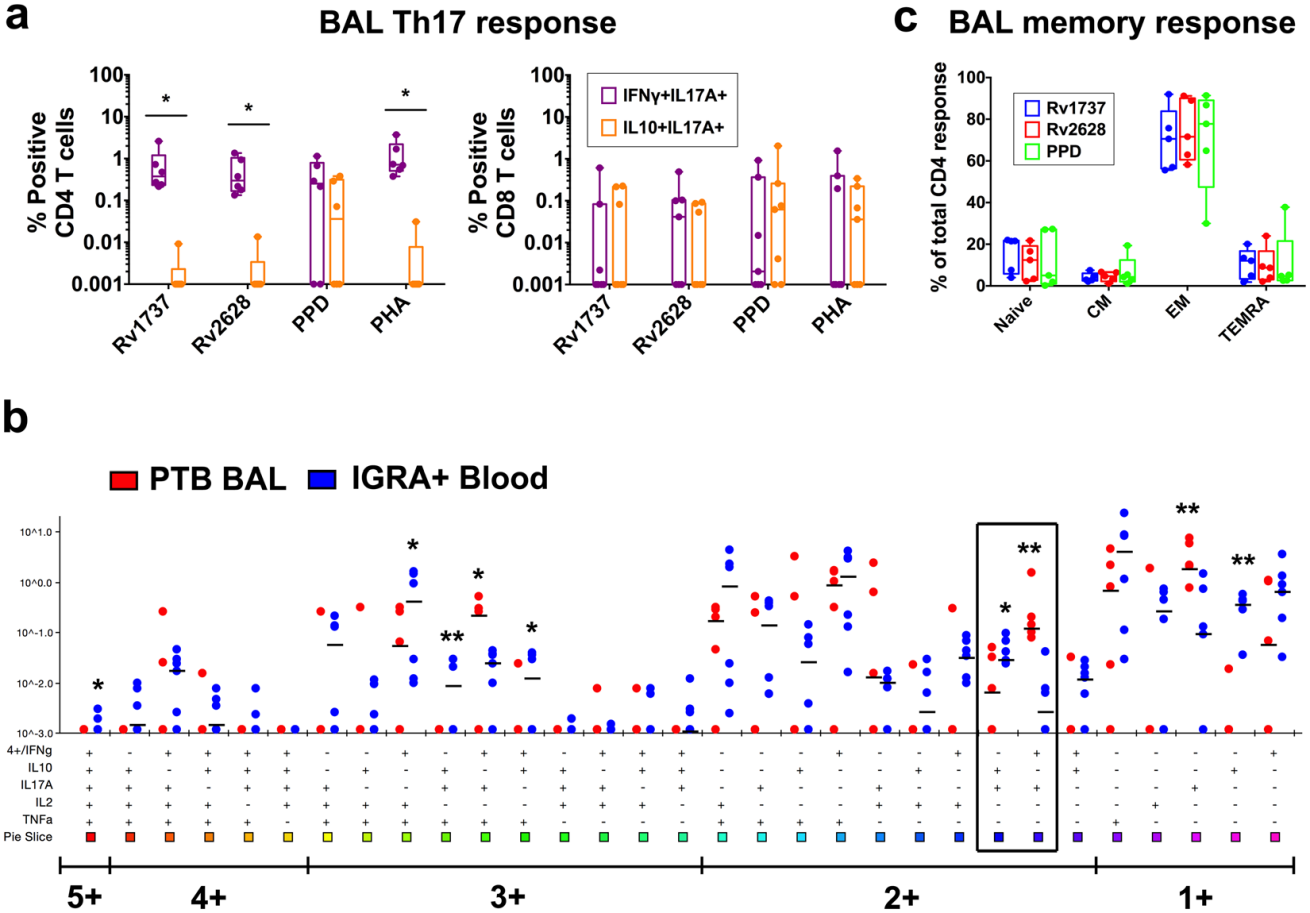
PTB patients have a proinflammatory Th17 and an effector memory response to latency antigens in the BAL. BAL cells from PTB subjects (N = 5) and PBMC from IGRA^+^ (N = 6) were cultured overnight in a standard ICS assay in the presence of Mtb DosR regulon encoded (Rv1737c & Rv2628) antigens or PPD or PHA. (**a**) Box-and-whisker plots show the range in frequencies with horizontal bar representing the median of individual Th17 subsets. Statistical differences between groups was calculated using Wilcoxon matched-pairs signed rank test: *p < 0.05. (**b**) Polyfunctionality of BAL CD4 T cells was compared to LTBI blood CD4 T cells. The box highlights the two important subsets IL10^+^/IL17A^+^ and IFNγ^+^/IL17A^+^ subsets. Statistical differences between groups was calculated by Mann-Whitney U test: *p < 0.05; **p < 0.01. (**c**) Box-and-whisker plots show the range in percentages of individual memory subsets with the horizontal bar within the box showing the median.

Mtb-specific T cells can be detected in bronchoalveolar lavage (BAL) fluid of PTB subjects^43^. We therefore tested if reduced blood latency antigen specific responses in PTB (Fig. 1) is due to accumulation of these cells at the site of infection by comparing BAL versus matched blood (see Supplementary Fig. 4 for gating strategy). CD3^+^ T-cells were reduced in BAL^44^, with 3 of 7 donors lacking CD3^+^ T-cells in BAL (Fig. 5a). The CD3^+^ CD4:CD8 T-cell ratio was also altered, with BAL CD4 but not CD8 percentages being significantly lower than blood (Fig. 5a). Conversely, there was a marked increase in CD3^+^CD4^−^CD8^−^ BAL T-cells, which could represent a mixed population of either conventional T cells that have down regulated CD4 and CD8 co-receptors or unconventional T cells (Fig. 5a). An identifying feature of BAL CD4^+^ and CD8^+^ T cells, but not the CD3 double negative population, was high spontaneous cytokine expression compared to either matched blood (Fig. 5b), or PBMC from IGRA^+^ and EPTB subjects (Supplementary Fig. 5a), indicating that BAL cells are inherently activated. Frequencies of Rv1737c and Rv2628 latency antigen specific BAL T cells was determined after subtraction of background spontaneous cytokine expression. Thus, antigen-induced IFNγ responses was very polarised in the BAL with significantly higher frequencies of latency antigen-specific CD4 T cells compared to matched blood detected only in donors who responded to stimulation (Fig. 5c), whereas responses to PPD was less marked and PHA being comparable. An overall similar pattern was also noted in the CD8 compartment with noticeably higher intragroup variation whilst the response of the CD3^+^CD4/CD8 double negative population in BAL and blood was comparable (Fig. 5c). Within the CD4 and CD8 antigen specific subsets, BAL but not matched blood CD4 T cells express IL17 (Supplementary Fig. 5b) to stimulation with latency antigens and mitogen, but not to PPD stimulation (consistent with Fig. 1 data). Polyfunctionality of PTB BAL T cells determined by SPICE were almost exclusively dominated by the IFNγ^+^/IL17^+^ subset and not IL10^+/^IL17^+^ CD4^+^ Th17 cells as in LTBI subjects, however no significant difference was observed in these subsets within the CD8 T cell compartment (Fig. 6a,b **)**. The composition of BAL CD4 T cells was dominated by CD4 effector rather than central memory cells (Fig. 6c). These data further confirm that Mtb latency antigen specific BAL CD4 T cells have a similar profile as cells of similar specificity in the blood of EPTB subjects. We conclude that Mtb latency antigen specific Th17 proinflammatory T cells are an important component of the overall T cell response in subjects with either pulmonary or extrapulmonary TB.

## Discussion

This study provides new and important insights into the nature of the T cell response in humans infected with Mtb. Although it is well recognised that pathogen specific immunity is influenced by pathogen load and site of infection, we provide unequivocal evidence for this in tuberculosis. Novel aspects of our work are: (i) that the magnitude of blood T cell responses in subjects with pulmonary and extrapulmonary TB differs significantly (Fig. 1); (ii) that there is significant functional diversity in Mtb T cell responses which is visualized in the response to Mtb latency, but not secretory antigens (Fig. 3); (iii) that polyfunctional responses are significantly higher in latent TB particularly in response to latency but not classical secretory antigens (Figs 3 and 4); (iv) that latent TB subjects induce an enhanced latency antigen specific regulatory IL10^+^ Th17 response, whereas subjects with either EPTB or PTB induce a specific Th17 response that is skewed to an inflammatory phenotype; (v) that the functional composition of Mtb specific T cells in pulmonary and extrapulmonary TB is remarkably similar except that specific T cells accumulate *in situ* in the lung in PTB, whilst in EPTB similar cells are noted in blood (Fig. 6). Taken together, we provide strong evidence that Mtb DosR latency but not secretory antigen specific responses clearly differentiate the different clinical states of TB, with latency antigen specific Th17 regulatory responses in particular differing between subjects with disease versus controlled infection. Latent TB can be heterogeneous, comprising both truly latent subjects and subjects predisposed to progress to disease^45^. Our data now provides a rational basis for future investigation to test how the functional marker of CD4 T cells we have identified varies in subjects at different clinical stages of latent TB.

Our study and others concur on the importance of central memory T cells in immunity to TB^46^. Previous reports have demonstrated that active TB patients exhibit a higher frequency of IFNγ^+^TNFα^+^ CD4^+^ and CD8^+^ T cells with a T_EM_ phenotype compared to latently infected and subjects cured of TB by anti-tuberculosis treatment (ATT) display a T_CM_ phenotype^37^. Consistent with these data we also demonstrate that LTBI subjects, unlike those with either EPTB or PTB, have significantly higher frequencies of central memory T cells, irrespective of the antigen tested (Fig. 2). Conversely subjects with active disease had significantly higher effector memory responses consistent with persistent antigenic stimulation likely linked to higher bacterial burden^47^.

A key novel observation of our study is the striking difference in the nature of the T cell response induced by the array of T cell antigens expressed by Mtb. The importance of Mtb DosR latency antigens in LTBI has been reported, but the functional diversity of the T cell response to these antigens in relation to clinical phenotypes and *in situ* responses (BAL vs. blood) has not yet been documented. Dormancy survival regulon (DosR) latency antigens are induced when Mtb encounters hypoxia, nutrient starvation, low nitric oxide or low pH that actually mimics the granuloma environment during latent infection^33,34^. Previous studies have shown that T cell responses to these antigens are predominantly present in subjects with latent as opposed to active TB, indicating a putative role in controlling progression of infection^35–37^. Our study highlights that a key feature of such a response may be linked to the ability to induce IL17 and IL10 in subjects with latent infection, but not those with disease. Precisely why latency but not secretory antigens induce IL17 is not known but may be linked to the capacity of these antigens to differentially induce other counterbalancing cytokines.

The dampening of peripheral blood responses during pulmonary TB may be explained in part by suboptimal activation of effector T cells as a result of downregulation of antigen-gene expression by Mtb^8^, or the egress of specific cells from blood or lymph nodes to the site of infection (the lung) and/or specific T cells resident in the lung being activated *in situ*. Conversely, the persistently elevated latency antigen-specific responses in blood observed in EPTB and LTBI may be due to continuous stimulation of antigen-specific CD4 T cells that efficiently recirculate between blood and tissues^48^, unlike cells from PTB. Whilst these possibilities remain to be fully explored, our comparative analysis of T cell responses in the broncheoalveolar lavage of PTB subjects versus matched blood cells, demonstrates that lack of blood responses in PTB does not reflect an overall failure to respond. Our findings corroborate with data reported by Chiacchio *et al*.^43^, showing higher frequencies of single IFNγ^+^ and IL2^+^ cells as well as double IFNγ^+^IL2^+^ cells in the BAL compared to PBMCs upon stimulation with Rv2628 and PPD. Other data highlight the importance of localised responses in TB control. Following Mtb infection, there is massive infiltration of lymphocytes to the lung which is necessary for granuloma formation and maintenance^34^. It has been suggested that the rapid accumulation of antigen-specific T cells in the lung after clonal expansion in lymphoid tissues probably plays an important role in mounting protective immunity during latent infection^49^. However, our observation that BAL T cells from PTB subjects have a functional Th17 profile that is similar to that seen in EPTB but differs from that seen in latent TB blood, demonstrates for the first time, that the cells accumulating in the lung of PTB subjects are likely skewed to a pathogenic rather than a protective functional phenotype.

A key question arising from our study is why Th17 responses differ between latent and active TB. Our results depict that IL17A is induced especially in response to latency but not secretory antigens and is higher in LTBI compared to PTB. This is consistent with previous studies showing Th17 cells to be protective during latent phase of infection while reduced IL17 levels during TB correlates with poor disease outcome^27,28^ and the proportion of pathogenic Th17 cells to directly correlate with clinical parameters associated with disease severity^29,30^. Additionally, an augmented Th17 response is associated with persistent and high antigen burden and drug resistance^50^. Moreover, infiltration of neutrophils to the diseased site is powered by IL17 that is known to exacerbate TB by inducing uncontrolled inflammation and T cell dysfunction^51^. A number of cell and molecular pathways can regulate IL17 expression^52–54^; one key mechanism may be linked to loss of IL6R on CD4^+^ T cells or suppression of IL17 by IL10 induced by upregulation of IL27 signaling^28^. This, and the mechanisms for altered Th17 differentiation into subsets with a phenotype characteristic of “pathogenic Th17” in EPTB versus “protective Th17” in LTBI, remain to be elucidated.

In summary, our study provides compelling evidence for the presence of polyfunctional, regulatory Th17, CD4 central memory T cells, specific for Mtb latency antigens to be a marker of latent infection. We further conclusively demonstrate for the first time that the nature of CD4 T cell responses to stage-specific Mtb antigens to be strikingly different; wherein DosR latency antigen-specific regulatory IL10^+^ Th17 cells in blood during latent infection skew towards a pathogenic IFNγ^+^ Th17 phenotype evident in BAL or blood of subjects with pulmonary or extrapulmonary TB respectively. Our data provides a strong, rational platform to test the importance of DosR-specific IL17^+^IL10^+^IFNγ^+^ CD45RA^−^CD27^+^ CD4^+^ central memory T cells as a correlate of protective immunity in TB.

## Methods

### Study population

This study was performed in accordance with the relevant guidelines and regulations stated in the Declaration of Helsinki and approved by the Ethical Review Committee of St. John’s Medical College Hospital, Bangalore, India (Ref no: 55/2015). 205 individuals were prospectively recruited between April 2015 and March 2017 and were included in this study only after obtaining written consent. Relevant clinical information was documented in a pro forma and 30 ml of peripheral blood was collected by venepuncture in ACD, EDTA or heparin tubes depending on the immune assays. Matched sputum samples and/or tissue samples were collected wherever possible. Clinical information of study participants are summarized in Table 1. Four clinical groups were studied as described below:

**Table 1:** Clinical details for individuals used in this study.

Group	N	Median Age (range)	Gender (M/F)	Disease site	Tests conducted			
					QFT	X-ray	Sputum/FNA/Biopsy AFB	GeneXpert MTB/RIF
IGRA^+^/LTBI	15	31 (25–50)	12 M/3 F	—	+ve	ND	ND	ND
PTB	27	36.5 (18–66)	22 M/5 F	Lung	ND	✓	✓(Sputum +ve)	✓
EPTB	19	28 (17–46)	10 M/9 F	Cervical LN, TB pleurisy Peritoneal TB, Endometrial TB, Intestinal TB Cold abscess	ND	✓	✓(Sputum −ve); ZN staining	✓
PTB (BAL)	7	34.5 (20–55)	7 M	Lung	ND	✓	✓(Sputum −ve/+ ve)	✓

#### IGRA^+^ subjects or latent TB infection (LTBI)

Healthcare workers of St. John’s National Academy of Health Sciences were recruited through internal calls highlighting the nature and importance of the study. Study subjects were classified as IGRA^+^ based on a standard QuantiFERON TB Gold In-tube test (Qiagen, Germany) results^55^. IGRA^+^ subjects were classified as LTBI if they had not received preventive/curative therapy for TB in the past. A total of 88 subjects were recruited and screened by the IGRA test, of which 26 were IGRA^+^. 15 IGRA^+^ subjects were included for the study of which 80% were males with a median age of 31 years (25–50). Healthcare workers in India do not undergo routine IGRA screening at the time of employment. Hence, ascertaining the duration for which our study subjects were IGRA^+^ was not possible.

#### Pulmonary TB

Subjects for this prospective study were enrolled from the Revised National Tuberculosis Program (RNTCP) clinic of St. John’s Medical College and Hospital. A diagnosis of pulmonary TB was ascertained by sputum smear microscopy and culture. Standard smear grading of 1^+^, 2^+^ and 3^+^ was used to ascertain the bacterial burden. Smear negative cases of pulmonary TB cases were diagnosed and classified by the treating clinician from plain chest radiographs as per the RNTCP standards. According to the RNTCP guidelines, chest radiograph and sputum culture are not necessary for TB diagnosis. Smear positivity is considered sufficient to make a diagnosis of pulmonary TB in India. All patients included in the study were smear positive and positive for TB by GeneXpert. Rifampin resistance by GeneXpert was an exclusion criterion. All subjects were treatment naive at the time of enrolment. Consenting adult patients meeting the above inclusion and exclusion criteria were included. Sputum samples were collected for reconfirmation of TB diagnosis by GeneXpert MTB/RIF assay (Cephid, USA). A total of 27 cases of pulmonary TB were included in the study of which 81.48% were males with a median age of 36.5 years (18–66).

#### Extrapulmonary TB

Patients attending the RNTCP clinic of St. John’s Medical College and Hospital were prospectively included in the study. A diagnosis of extrapulmonary TB was established from tissue specimens by ZN staining for detection of AFB in tissue samples (obtained as surgical specimens/biopsies/FNACs). The RNTCP approved the GeneXpert MTB/RIF assay which is a nucleic-acid amplification (NAA) test of tissue sample for confirmation of diagnosis. The site of extrapulmonary TB varied: 13 cases had cervical lymphadenitis and 2 had tubercular pleural effusions while the remaining four cases included one each of peritoneal TB, endometrial TB, intestinal TB and cold abscess. Among the total 19 extrapulmonary TB cases, 52.6% were males with a median age of 28 years (17–46).

### Bronchoalveolar lavage (BAL) collection & processing

Patients were enrolled at the outpatient department of the Department of Pulmonary Medicine and study was approved by the Ethical Review Committee of St. John’s Medical College Hospital, Bangalore, India (Ref no: 272/2015). Sputum negative cases with clinico-radiological suspicion of pulmonary TB were identified and BAL was isolated from these patients to confirm the preliminary diagnosis of TB. Patients with Chest X-ray suggestive of TB but sputum negative underwent bronchoscopy. BAL was checked for AFB and TB by GeneXpert and only those positive for these tests were included. Total number consented was 8; of which BAL samples from 7 subjects were analysed for this study (Table 1). The study subjects were asked to fast overnight (with a minimum fasting duration of 4 hr). A written consent was sought, prior to the start of the procedure. A viscous Xylocaine gargle was given to the study subjects and the vocal chords were sprayed with 2% Xylocaine. The region of interest (opaque lesion or cavitary lesion) was confirmed based on a plain chest radiograph or by CT thorax. Fiberoptic bronchoscope (Olympus, Japan) was then passed through one of the nostrils and the tracheobronchial tree was visualized. The bronchoscope was then passed until its tip was wedged in the region of interest. 50 ml of sterile isotonic saline solution was then introduced through the working channel of the bronchoscope using a 25 ml syringe. The lavage was then aspirated out using the same syringe with general suction, collected in sterile 50 ml centrifuge tubes containing X-VIVO media (Lonza, Switzerland) supplemented with 5% human AB serum (GemCell, US) and immediately processed for cell isolation^56^. BAL cells obtained after centrifugation were filtered using 40μm cell strainer (Falcon, Fischer scientific) in order to obtain uniform single-cell suspension.

### Antigens and Antibodies

4 DosR regulon-encoded latency antigens (Rv1733c, Rv1737c, Rv2029 and Rv2628) and 3 secretory Mtb antigens (Ag85A/B, TB10.4, ESAT6/CFP10) were synthesized as recombinant proteins at the Department of Infectious Diseases, Leiden University Medical Center. CMVpp65 and CandidaMP65 peptide pools were procured from JPT Peptide Technologies (Berlin, Germany). Phytohemagglutinin (PHA) was purchased from Remel, Thermo Fisher Scientific and Staphylococcus enterotoxin B (SEB) was purchased from Sigma-Aldrich. Purified protein derivative (PPD) was procured from Staten Serum Institute, Denmark. Fluorochrome conjugated monoclonal antibodies to cell surface and intracellular markers used in flow cytometry assays are listed: CD45RA-APC-H7 (HI100), CD27-BV785 (O323), CCR7-Alexa Fluor 647 (G043H7), γδTCR-PE-CF594 (B1), CD3-BV570 (UCHT1), CD4-BUV395 (SK3), CD8-BV711 (RPA-T8), IFNγ-V450 (B27), TNFα-FITC (MAb11), IL2-APC or IL2-Alexa Fluor 700 (MQ1-17H12), IL17A-BV605 (BL168), IL17F-BV650 (O33-782), IL10-BV786 (JES3-9D7), IL22-PE-Cy7 (22URTI), and MIP1β-PE (D21-1351). Antibodies were sourced from BD Biosciences, BioLegend and eBioscience.

### Whole Blood Assay (WBA) for quantification of secreted cytokines

Analysis of immune responses to Mtb antigens or peptides was determined by measurement of cytokine production post 7 day antigenic stimulation of whole blood^57^. Briefly, lyophilized DosR latency antigens were reconstituted in sterile 1x PBS and diluted to a final concentration of 20 µg/ml with RPMI 1640 containing L-glutamine. The diluted antigens (100 µl at 20 µg/ml) as well as the medium (unstimulated control), were then seeded into 96-well round-bottom microtiter plates in triplicates after which plates were frozen at −80 °C. On the day of WBA, within 3 hr of blood draw, heparinized whole blood from IGRA^+^(LTBI) individuals and infected PTB and EPTB patients was diluted 1:5 in pre-warmed (37 °C) RPMI-1640 medium containing glutamine and 100 μl of blood/well was transferred to plates that were previously loaded with antigens. TB-specific antigens Ag85A/B, TB10.4 and ESAT6/CFP10 fusion protein were added at a final concentration of 10 μg/ml. PPD (5 μg/ml), PHA (3 μg/ml) and culture medium (RPMI 1640) were used as positive controls and negative control respectively. Plates were incubated in a 5% CO_2_ incubator at 37 °C for 7 days, and culture supernatants were harvested and stored at −80 °C for further analysis.

### IFNγ ELISA

Supernatants were analysed by IFN-γ ELISA as previously described^58^. Briefly, plates were coated overnight with coating antibody (anti-human IFN-γ) at 4 °C. Following washing with PBS containing 0.05% Tween 20, wells were blocked at RT using assay diluent (PBS with 10% FCS) for 1 hr. Samples, controls and standards were then added and the plate incubated for 2 hr at RT. After washing, detection antibody (biotin anti-human IFNγ) + Streptavidin-HRP reagent was added, plates incubated for 1 hr at RT. Next, plates were washed and incubated with substrate solution containing Tetramethylbenzidine (TMB) and Hydrogen Peroxide for 30 min at RT in the dark. The reaction was stopped with 1 M H_3_PO_4_ and the plates were read at 450 nm. IFNγ levels were measured according to manufacturer’s instructions using the OptEIA™Human IFNγ ELISA set (BD Biosciences, UK).

### Intracellular cytokine staining (ICS) assay and multiparameter flow cytometry

ICS assay with antigen-stimulated PBMCs was performed as previously described^59^. PBMCs were isolated by density centrifugation (Histopaque) following manufacturer’s instructions. Cryopreserved PBMCs were taken out in ice and rapidly thawed in 37 °C water bath, transferred to 15 ml tube containing ~3 ml PBS and centrifuged at 2000 rpm for 5 min at RT. 1 × 10^6^ cells were resuspended in 200 µL medium [RPMI-1640 (GIBCO, Invitrogen) with 10% FCS (GIBCO), 100 U/ml penicillin, 100 µg/ml streptomycin, (SIGMA)], seeded in 96-well round-bottom plates (Costar) and stimulated with different recombinant DosR regulon-encoded antigens (10 µg/ml), secretory antigens (10 µg/ml), recall antigens CMVpp65 (1.7 µg/ml) and CandidaMP65 (1 µg/ml) peptide pools and SEB (1 µg/ml) at 37 °C and 5% CO_2_. After 4 hr of incubation, brefeldin A (10 µg/ml) (Sigma-Aldrich) was added. Next day, PBMC were washed after incubation with EDTA and stained with 5 μl Live/Dead Aqua (Invitrogen). Next, 50 μl of cell surface staining cocktail was added and incubated for 30 min at RT in the dark. Cells were then fixed for 20 min with 100 μl 1X FACS lysis buffer and permeabilized with 200 μl 1X BD Perm/Wash buffer for 20 min. PBMCs were washed and incubated for 30 min at RT in the dark with 50 μl of intracellular staining cocktail. Cells were washed with 150 μl Perm/Wash buffer and resuspended in ~100 μl 1% paraformaldehyde.

#### ICS assay on BAL samples

BAL cells isolated as mentioned above were resuspended in X-VIVO media and cultured overnight with different antigens, stained and processed as mentioned above in PBMC ICS assay and acquired on flow cytometer^60^.

### Flow cytometry analysis

Samples were acquired on BD FACSAria^TM^ Fusion flow cytometer and BD FACSDiva^TM^ version 8.0.1 software (BD Biosciences). Cytometer Setting and Tracking (CST) beads (BD Biosciences) were acquired before each experiment to ensure that cytometer parameters remained consistent across all experiments. Stained samples were acquired with a standard stopping gate set at 100,000 CD3 lymphocytes. Negative and single-stained compensation beads (eBioscience) were acquired for each experiment, before sample acquisition, and used to calculate the compensation matrix. Data was analysed using FlowJo version 9.9.4 software (Treestar) as well as Pestle v1.8 and SPICE (Simplified Presentation of Incredibly Complex Evaluations) v5.1^42^.

### COMPASS analysis of flow cytometry data

Cell counts were analysed using the COMbinatorial Polyfunctionality Analysis of Antigen-Specific T cell Subsets (COMPASS) algorithm as described in^40^. Briefly, COMPASS is a statistical model developed for high-dimensional flow cytometry data analysis that can detect antigen-specific changes across all observable functional T cell subsets, without the need to limit the analysis to very specific subsets based on expected biological significance. In COMPASS, responses are quantified using posterior probabilities that summarize for each subject and subset the evidence that the corresponding response is Ag specific by comparing the proportion of cytokine-positive cells in the Ag sample to the corresponding proportion in the control sample. Polyfunctionality scores were calculated from posterior responses probabilities, summarizing a subject’s entire T-cell functionality profile into a single number. In this study, we have applied COMPASS to each of the 10 antigens in 2 (CD4^+^ and CD8^+^) T cell subsets leading to 20 analyses. Each one of the analyses was unbiased and considered all of the 31 possible cytokine functions (defined as Boolean combination). In order to evaluate differences in polyfunctionality between groups, a linear model estimating the group wise mean polyfunctionality scores was fit to each antigen and the difference between IGRA^+^ and other groups was tested (Wald test, null: ${\hat{\beta }}_{group}-\,{\hat{\beta }}_{IGRA+}=\,0$, two-sided test). Resulting p-values were adjusted for multiple testing (across all 60 tests and models) to control the FDR (false discovery rate) using the method of Benjamini & Hochberg. Significant differences were called at the 5% FDR level. Magnitudes of T cell responses were calculated independent of COMPASS as the maximum of zero or the proportion of gated events in the stimulated condition minus the proportion of gated events in the unstimulated condition.

### Statistical analysis

All antigen-stimulated wells were adjusted for non-specific responses by background subtraction (media alone). A Kruskal-Wallis test followed by Dunn’s post-test comparison was used for determining differences between LTBI (IGRA^+^) subjects and PTB and EPTB cases. An unpaired two-sided Mann–Whitney U-test was used for analysis when comparison was made between the clinical groups and a two-sided Wilcoxon matched pairs signed rank test was performed when comparing responses to BAL versus matched blood. Analyses were performed using Graphpad Prism 6 (Software MacKiev, USA). Significant differences are indicated: *p < 0.05; **p < 0.01; ***p < 0.001; ****p < 0.0001.

### Data availability statement

Supporting Data is attached.

## Electronic supplementary material

Supplementary Information

Supplementary Dataset
