# Supplementary material for: Circulating Mycobacterium tuberculosis DosR latency antigen-specific, polyfunctional, regulatory IL10+ Th17 CD4 T-cells differentiate latent from active tuberculosis
Source: Sci Rep. 2017 Sep 20;7:11948. doi: 10.1038/s41598-017-10773-5 (PMC5607261; doi:10.1038/s41598-017-10773-5)
Supplement: Supplementary file 1 — Supplementary Information [file 41598_2017_10773_MOESM1_ESM.pdf]

SUPPLEMENTARY MATERIAL

Circulating *Mycobacterium tuberculosis* DosR latency antigen-specific, polyfunctional, regulatory IL10^{+} Th17 CD4 T-cells differentiate latent from active tuberculosis.

Srabanti Rakshit[1], Vasista Adiga[1], Soumya Nayak[1], Pravat Nalini Sahoo[1], Prabhat Kumar Sharma[1], Krista E. van Meijgaarden[2], Anto Jesuraj UK J[3], Chirag Dhar[3], George D Souza[4], Greg Finak[5], Stephen C. De Rosa[5,6], Tom H. M. Ottenhoff[2], Annapurna Vyakarnam[1,7*]

[1]Laboratory of Immunology of HIV-TB co-infection, Centre for Infectious Disease Research, Indian Institute of Science, Bangalore, India.

[2]Department of Infectious Diseases, Leiden University Medical Center, Leiden, The Netherlands.

[3]Department of Infectious Diseases, St John's Research Institute, Bangalore, India.

[4]Department of Pulmonary Medicine & Department of Infectious Diseases, St John's Research Institute, Bangalore, India.

[5]Vaccine and Infectious Disease Division, Fred Hutchinson Cancer Research Center, Seattle, WA, United States of America.

[6]Department of Laboratory Medicine, University of Washington, Seattle, WA, United States of America.

[7]Department of Infectious Diseases, King's College London, Guy's Hospital, London SE1 9RT, United Kingdom.

***Correspondence to:**

Dr A Vyakarnam
Centre for Infectious Disease Research
Indian Institute of Science
Bangalore 560012
Tel: 0091-80-2360-4348 (land line)/0091-9945385960 (mobile)
E-mail: anna.vyakarnam@kcl.ac.uk
annapurnavyakarnam@gmail.com

Supplementary Figure Legends

Supplementary Figure 1: Gating strategy to identify cytokine secreting CD4 and CD8 T cells. Representative data of PBMCs from Rv1737c-stimulated IGRA^{+} individual is shown. Sequential gating included: (A) Singlets were gated using FSC-A and FSC-H; (B) live Avid^{low} cells (C) gating on FSC-A and SSC-A to discriminate Lymphocytes. (D) CD3^{+} cells; (E) CD4^{+} and CD8^{+} T cells. (F and G) Quadrant gating on CD4^{+} and CD8^{+} T cells based on CD45RA and CD27 expression: naïve, T_{CM}, T_{EM} and TEMRA; region gates identify single and double positive populations for: IFNγ vs IL2 (H & I), IFNγ vs TNFα (J & K), IFNγ vs IL17A (L & M) and IFNγ vs MIP1β (N & O) respectively.

Supplementary Figure 2: CD8 T cell frequencies to Mtb-specific and common recall antigens. PBMCs from subjects with LTBI, PTB and EPTB (N=6) were cultured overnight in a standard ICS assay in the presence or absence of (a) secretory (Ag85A/B, TB10.4, ESAT6/CFP10 & PPD), (b) DosR (Rv1733c, Rv1737c, Rv2029 & Rv2628) and (c) common recall (CMVpp65 & CandidaMP65) antigens. CD3^{+}CD8^{+} T cells were analysed for intracellular expression of IFNγ, TNFα, IL2, IL17A and MIP1β. Box-and-whisker plots show the range in frequencies of total cytokine-positive CD8^{+} T cells with the horizontal bar within the box showing the median. One-Way ANOVA was used to determine between group statistical significance: *p<0.05; **p<0.01; ***p<0.001.

Supplementtray Figure 3: Comparison of regulatory IL10^{+} and proinflammatory IFNγ^{+} Th17 responses in IGRA^{+} and EPTB subjects to individual DosR latency antigens analyzed by COMPASS and SPICE. PBMCs from subjects with LTBI and EPTB (N=10) were cultured overnight in a standard ICS assay in the presence or absence of DosR (Rv1733c, Rv1737c, Rv2029 & Rv2628) antigens. CD3^{+}CD4^{+} T cells were analysed for intracellular expression of IFNγ, IL17A, IL17F, IL22, IL10, TNFα, IL2 and MIP1β. Boolean gates were created from the 8 individual cytokine (listed above) gates in FlowJo to divide responding cells into 256 distinct subsets corresponding to all possible combinations of these functions. These were analysed using COMPASS or SPICE software. (a) Boxplots of data is shown for the EPTB and IGRA^{+} groups of probability scores of antigen-specific response for each subject for each DosR antigen, averaged across regulatory or proinflammatory Th17 functional profiles, either IL10^{+} or IFNγ^{+} cells together with all combinations of IL17A, IL17F and IL22 respectively. Probabilities are derived from a linear model fit to ICS data using the Th17 panel. A p-value is shown testing for a difference between groups (two-sided t-test). (b) Box-and-whisker plots show the range in frequencies with horizontal bar

representing the median of regulatory Th17 subsets ($IL10^+IL17A^+$, $IL10^+IL17F^+$ and $IL10^+IL22^+$) on the left panel and proinflammatory Th17 subsets ($IFN\gamma^+IL17A^+$, $IFN\gamma^+IL17F^+$ and $IFN\gamma^+IL22^+$) on the right panel, induced by individual DosR antigen stimulation analysed by SPICE. Statistical differences between groups was calculated by Mann-Whitney U test: *$p<0.05$; **$p<0.01$; ***$p<0.001$.

Supplementary Figure 4: Gating strategy to identify cytokine secreting immune cells in BAL. Representative data of freshly isolated unstimulated BAL from a PTB is shown. Sequential gating included: (A) Lymphocytes based on FSC-A and SSC-A; (B) singlets (based on FSC-A and FSC-H; (C) Live ($Avid^{low}$) cells; (D) $CD3^+$ cells; (E & F) $CD4^+$, $CD8^+$ and $CD4^-CD8^-$ T cells. Quadrant gating on $CD4^+$, $CD8^+$ and $CD4^-CD8^-$ cells represent single and double positive populations for; IFNγ vs IL17A (G, H, I, J); IFNγ vs TNFα (K,L, M & N); IFNγ vs MIP1β (0, P Q& R); IFNγ vs IL10 (S, T, U & V); and IFNγ vs IL2 (W, X, Y & Z) respectively.

Supplementary Figure 5: Comparison of spontaneous cytokine expression in blood from diverse clinical groups versus BAL from PTB. (a) Box-and-whisker plots show the range in frequencies (horizontal bar within the box showing the median) of spontaneous cytokine expressing cells in BAL (pink) and matched PBMC (red) of PTB patients along with blood responses in EPTB (green) and $IGRA^+$ (blue) subjects. N=7 for BAL and N=12 for $IGRA^+$, PTB and EPTB. One-Way ANOVA was used to determine statistical significance between groups and corrected for multiple comparisons using Bonferroni's test. ***$p<0.001$; ****$p<0.001$. (b) Box-and-whisker plots show the range in frequencies with horizontal bar representing the median of antigen-induced IL17A expressing cells (after subtracting spontaneous release) in BAL (blue) and matched PBMC (red).

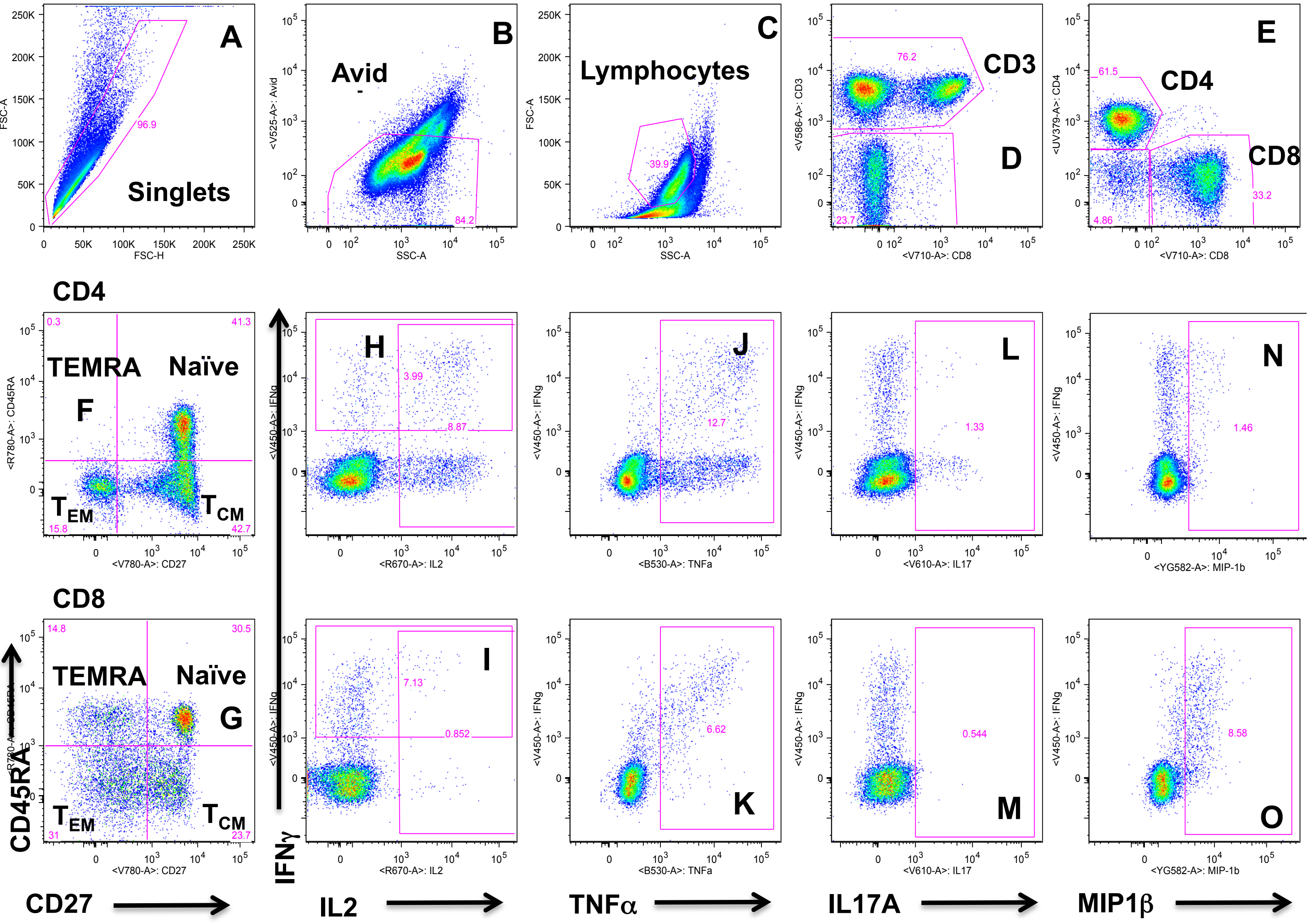

Supplementary Figure 1

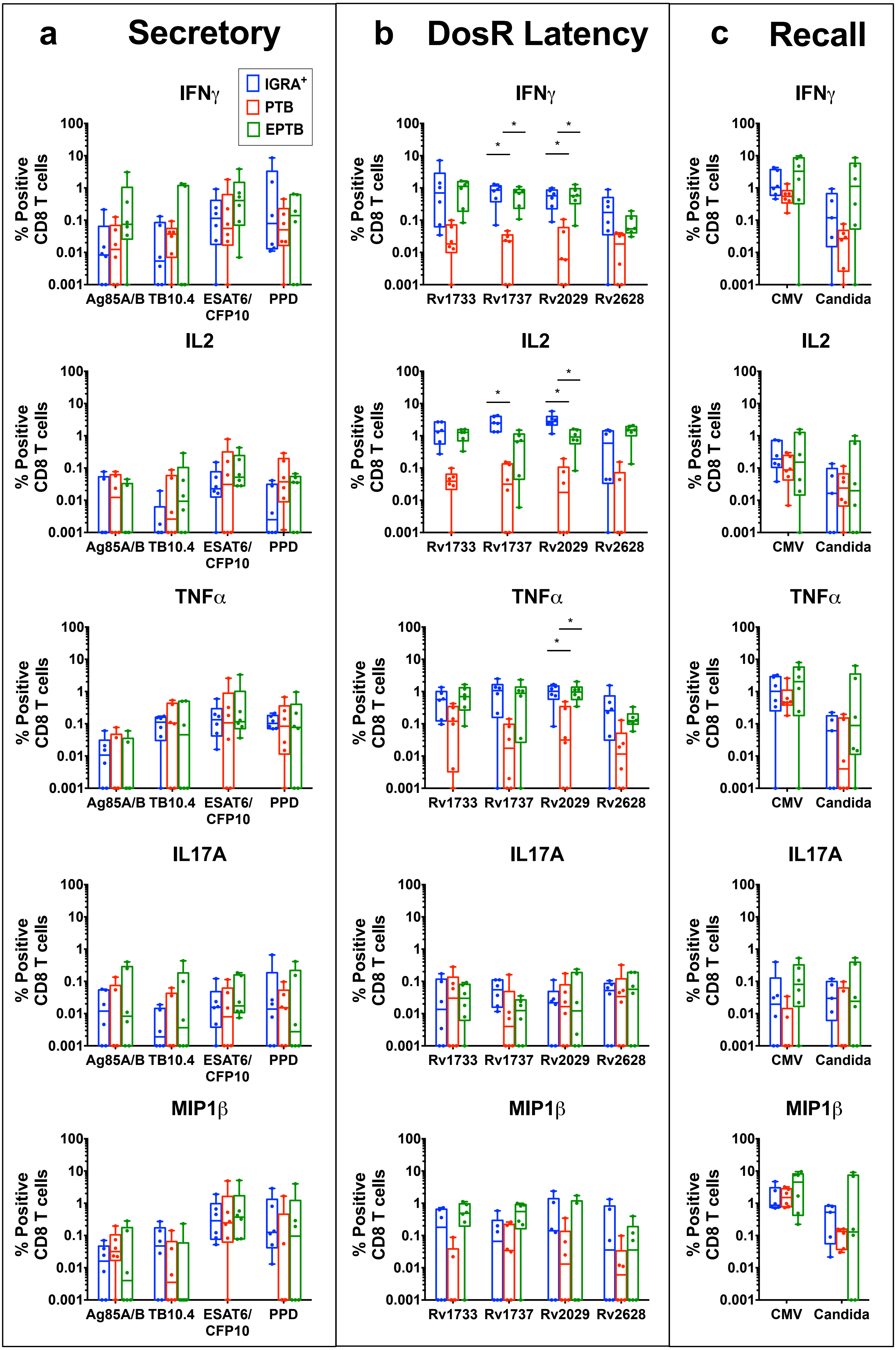

Supplementary Figure 2

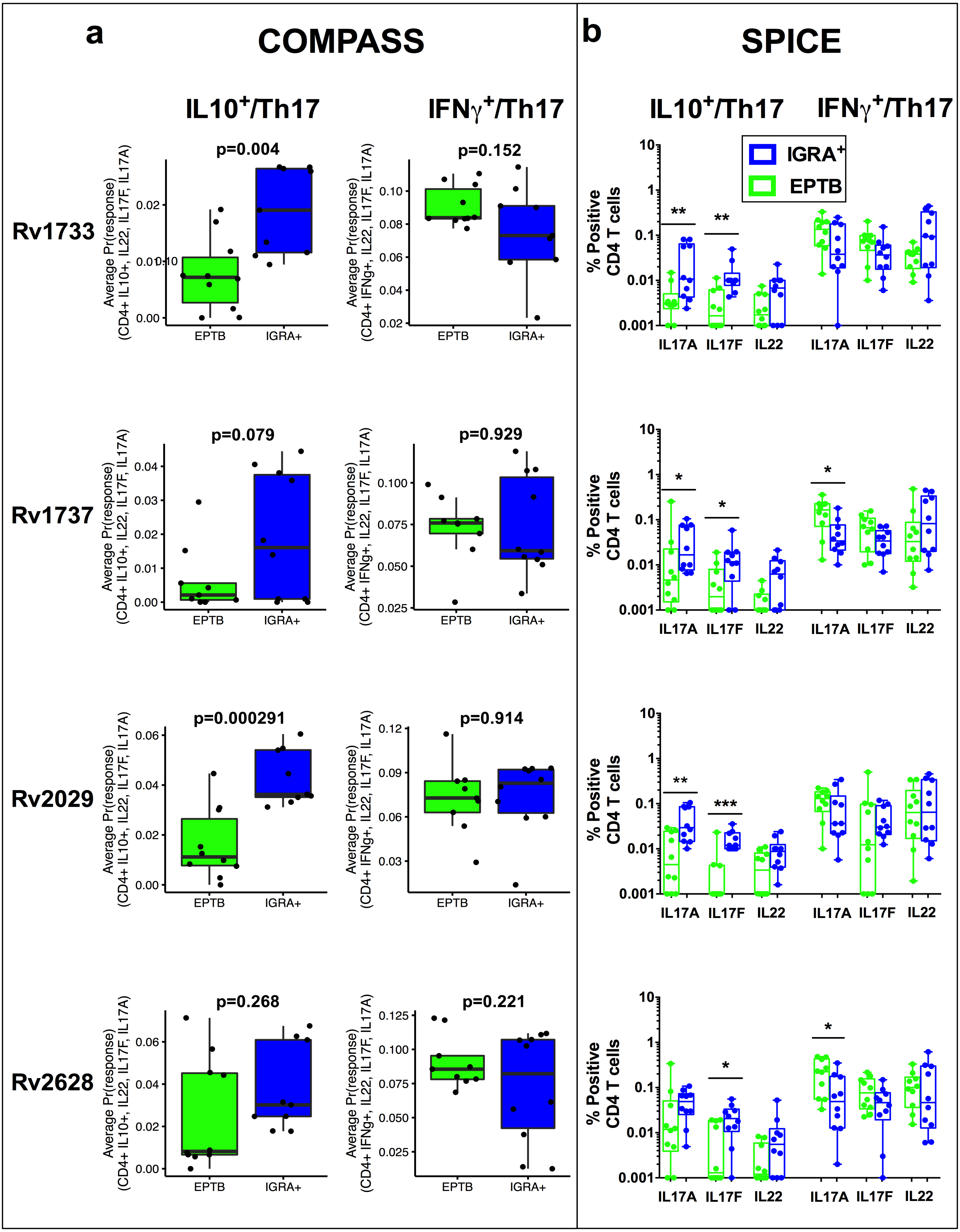

Supplementary Figure 3

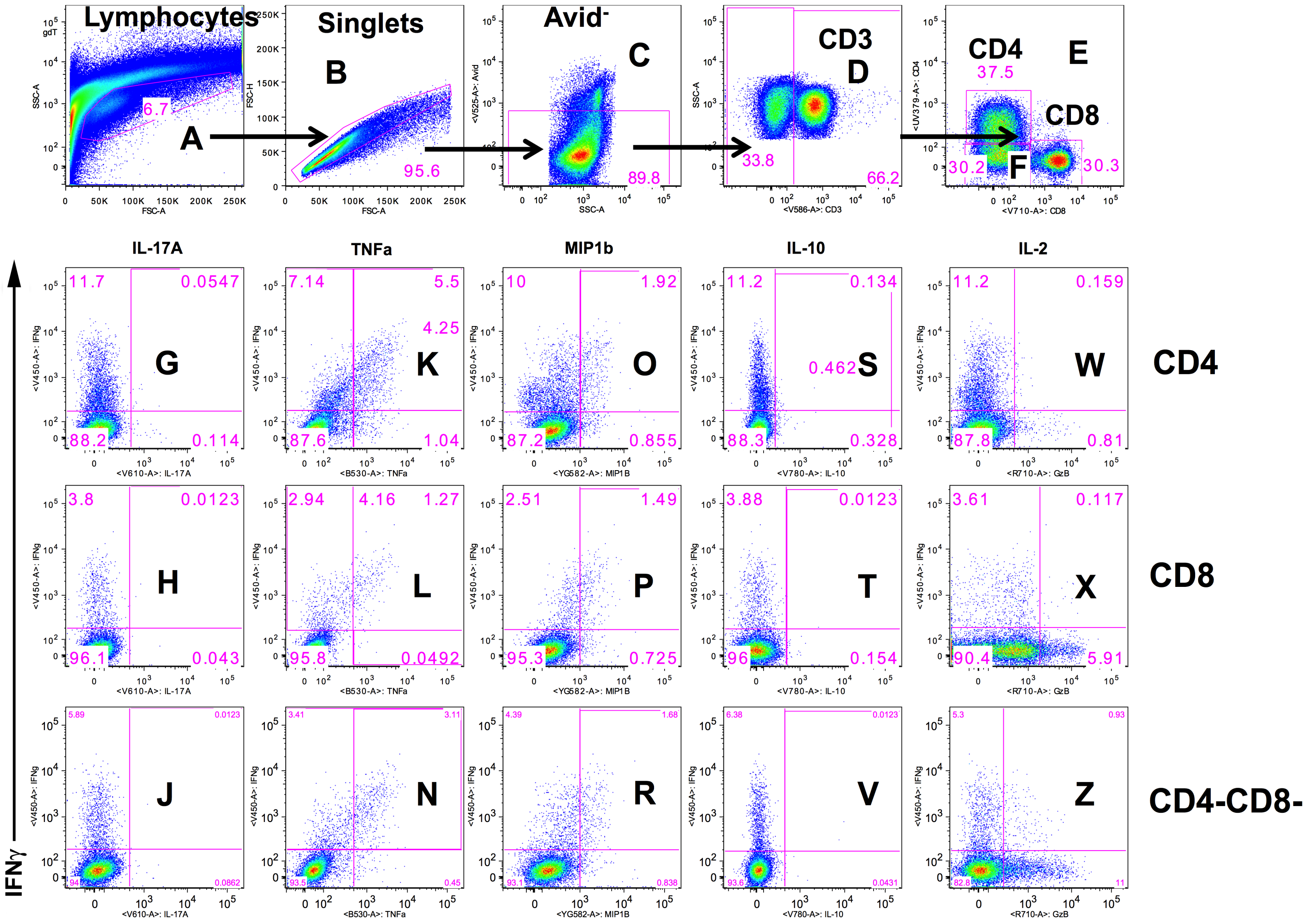

Supplementary Figure 4

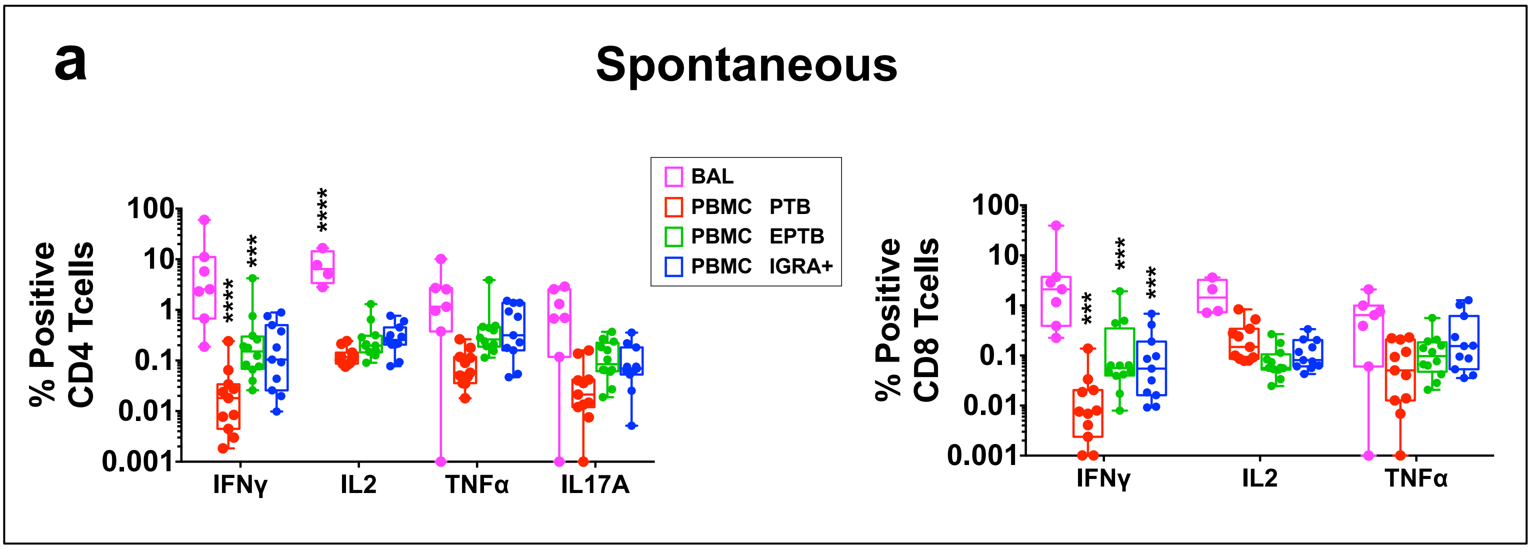

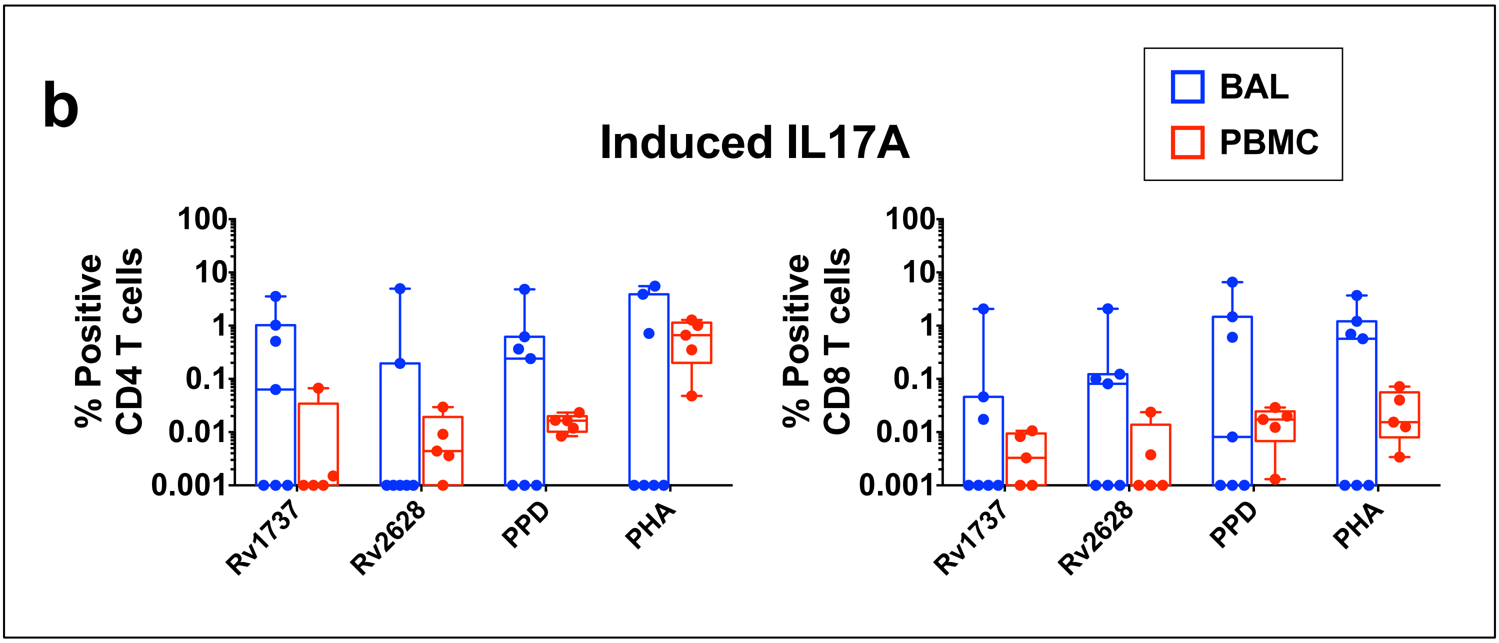

Supplementary Figure 5
